# Metagenomic Analysis of Microbial Community Compositions and Cold-Responsive Stress Genes in Selected Antarctic Lacustrine and Soil Ecosystems

**DOI:** 10.3390/life8030029

**Published:** 2018-07-11

**Authors:** Hyunmin Koo, Joseph A. Hakim, Casey D. Morrow, Michael R. Crowley, Dale T. Andersen, Asim K. Bej

**Affiliations:** 1 Department of Biology, University of Alabama at Birmingham, Birmingham, AL 35294, USA; joe21@uab.edu; 2 Department of Cell, Developmental and Integrative Biology, School of Medicine, University of Alabama at Birmingham, Birmingham, AL 35294, USA; caseym@uab.edu; 3 Department of Genetics, Heflin Center Genomics Core, School of Medicine, University of Alabama at Birmingham, Birmingham, AL 35294, USA; mcrowley@uab.edu; 4 Carl Sagan Center, SETI Institute, Mountain View, California, CA 94043, USA; dandersen@seti.org

**Keywords:** MG-RAST, cold shock, Lake Untersee, Ace Lake, Newcomb Bay Lake, Mount Seuss, McMurdo Dry Valleys

## Abstract

This study describes microbial community compositions, and various cold-responsive stress genes, encompassing cold-induced proteins (CIPs) and cold-associated general stress-responsive proteins (CASPs) in selected Antarctic lake water, sediment, and soil metagenomes. Overall, Proteobacteria and Bacteroidetes were the major taxa in all metagenomes. *Prochlorococcus* and *Thiomicrospira* were highly abundant in waters, while *Myxococcus*, *Anaeromyxobacter*, *Haliangium,* and *Gloeobacter* were dominant in the soil and lake sediment metagenomes. Among CIPs, genes necessary for DNA replication, translation initiation, and transcription termination were highly abundant in all metagenomes. However, genes for fatty acid desaturase (FAD) and trehalose synthase (TS) were common in the soil and lake sediment metagenomes. Interestingly, the Lake Untersee water and sediment metagenome samples contained histone-like nucleoid structuring protein (H-NS) and all genes for CIPs. As for the CASPs, high abundances of a wide range of genes for cryo- and osmo-protectants (glutamate, glycine, choline, and betaine) were identified in all metagenomes. However, genes for exopolysaccharide biosynthesis were dominant in Lake Untersee water, sediment, and other soil metagenomes. The results from this study indicate that although diverse microbial communities are present in various metagenomes, they share common cold-responsive stress genes necessary for their survival and sustenance in the extreme Antarctic conditions.

## 1. Introduction

The geophysical transformation accompanied by climatic changes during the Earth’s evolution for the last ~4 billion years resulted in the establishment of a wide range of ecosystems on this planet, and various lines of evidence suggest that microbial life has existed and evolved during the last ~3.5 billion years of Earth’s history [1,2]. These ecosystems, diverse in their physicochemical properties and changing over time, have required that life develop novel metabolic strategies for the exploitation of the numerous niches available across the planet. The functioning of microorganisms at the cellular and community levels is constrained by a set of physicochemical parameters within the ecosystems they inhabit [3]. Some of these ecosystems are categorized as “extreme habitats” where the inhabiting organisms have adapted to environmental parameters often considered inimical to the maintenance of life-functions for others [4]. Given that over 70% of Earth maintains near- or below-freezing temperatures, the cold ecospheres constitute the largest “physical extremes” for microbial communities to inhabit, manifest adaptive attributes, and drive key biological and geochemical processes [5,6].

Within the cryosphere, the Antarctic continent offers perennially cold, subzero temperatures as well as other challenges, such as oligotrophy, intense winds, aridity, and high solar UV radiation (during the austral summer months). Thus, all organisms, including microorganisms, living on this icy continent must possess various adaptive traits to sustain life [7]. Early studies of soil microbiology in the McMurdo Dry Valleys relied upon culture-dependent methods [8], however the diversity and distribution of microorganisms in various Antarctic ecosystems were not more fully explored until the advent of DNA-based culture-independent methods [5,6,7,9,10,11,12,13,14,15]. Recently, the applications of shotgun sequencing of the metagenome have enabled the elucidation of both taxonomic identities as well as crucial adaptive genetic traits necessary for microorganisms to cope with the environmentally imposed physical and nutritional extremes on this icy continent [16,17,18,19,20,21].

Although about 98% of the Antarctic landmass is covered by an ice sheet with a mean thickness of 2.16 km (maximum thickness up to ~4.78 km), a number of ice-free “oases” exist where various open-water lakes, perennially-ice covered lakes, and ponds largely support life [22,23]. Among these lakes, the perennially ice-covered lakes are particularly interesting to explore microbial communities and their adaptive strategies due to their unique physical, chemical, and limnological features [24]. The permanent ice cover (~3–5 m) of these lakes restricts wind-driven mixing of the water column, exchange of atmospheric gases, deposition of sediments, and light penetration [25,26]. In addition, most of these lakes manifest a stable water column with strong chemical stratification and minimal vertical mixing [27,28]. Lake Untersee is one of the largest (11.4 km^2^) and deepest (>160 m) perennially ice-covered ultraoligotrophic freshwater lakes in Antarctica. This lake is located in the Grüber Mountains of Central Queen Maud Land in East Antarctica and is partly dammed by the Anuchin Glacier [29,30,31,32]. The water column in Lake Untersee is well-mixed due to the temperature gradient (~0 °C on the top and 4 °C at the bottom) [29], contains a high concentration (150%) of dissolved oxygen, and harbors benthic photosynthetic microbial mats [30]. In addition, the high pH gradient, ranging between 9.8 and 12.1, unusual dynamics of temperature and water circulation, high methane content in some locations, and ultraoligotrophic conditions offer unique challenges to the microbial communities in this lake [29,30,33].

In this study, we have used the shotgun metagenomics approach along with bioinformatics tools to explore the microbial community compositions and genetic signatures for cold-responsive genes that code for cold-induced proteins (CIPs) and cold-associated general stress-responsive proteins (CASPs) in microbial communities of Lake Untersee water and sediment samples. In addition, we have used seven publicly available shotgun metagenome datasets of various other Antarctic lake and soil samples to compare and contrast the metagenomic profile of microbial communities and cold-responsive stress genes in Lake Untersee water and sediment samples.

## 2. Materials and Methods

### 2.1. Sample Collection

**Figure 1:**
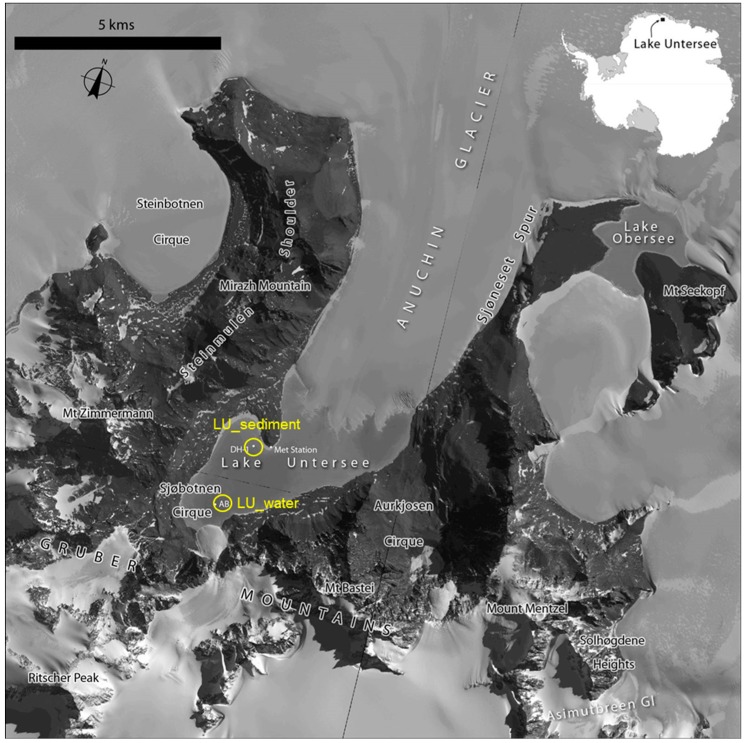
Satellite image map of Lake Untersee. Satellite imagery copyright DigitalGlobe, Inc. and provided by the NGA Commercial Imagery Program. The locations for the Lake Untersee water (LU_water) and sediment (LU_sediment) metagenomes are shown (circles).

Lake Untersee water samples were collected from the south basin at a depth of 80 m (71.35609° S, 13.4268° E) (Figure 1) using a 2.2 L acrylic Kemmerer bottle (Wildco, Yulee, FL, USA) via 25 cm holes drilled through the ice-cover. These samples were then filtered using cellulose nitrate membrane filters (Whatman, 47 mm × 0.2 μm) (membrane filters herein) to obtain cells for DNA extraction. The lake sediment samples were collected at a depth of 15 m (71.34197° S, 13.45458° E) by inserting 50- or 100 mm diameter polycarbonate core tubes into the lake floor, gently removing them without disturbance, sealing them with rubber stoppers, and then returning them to the surface. Samples were then divided into 1 cm sections, preserved, and stored as stated above. The collection of sediment samples was conducted by scientific divers via a dive hole (71.34197° S, 13.45458° E) on the lake using the techniques developed for studies of Antarctic lakes [34]. Immediately after collection, the water and sediment samples were preserved in 70% ethanol, first at −20 °C and then at −80 °C, at the Antarctic Logistics Centre International (ALCI), Cape Town, South Africa facility. Then, samples were transported to University of Alabama at Birmingham (UAB) in dry ice and kept at −80 °C until further use.

A total of seven publicly available Antarctic metagenome datasets were downloaded from the Metagenomics Analysis Server (MG-RAST) [35,36] and then used to filter the CIPs and CASPs. The metagenome dataset used in our study was derived from (1) water metagenomes from Ace Lake (*n* = 1) and Newcomb Bay Lake (*n* = 1); and (2) soil metagenomes from Mount Seuss (*n* = 1) and McMurdo Dry Valleys (*n* = 4). Out of the four McMurdo Dry Valleys metagenomes, three were from the Taylor Valley floor adjacent to Lake Hoare, Lake Bonney, and Lake Fryxell, whereas the fourth sample was from Wright Valley. For simplicity of the data analysis and description, we have used the following designation of the metagenomes: AL_water for Ace Lake water; NB_water for Newcomb Bay Lake water; MS_soil for Mount Seuss soil; and MDV_soil for McMurdo Dry Valleys soil (Table 1). For Lake Untersee water (*n* = 1) and sediment (*n* = 1) metagenomes, we have used LU_water and LU_sediment, respectively.

### 2.2. DNA Extraction and Sequencing

Purification of community DNA from LU_water and LU_sediment samples was carried out by using sterilized scalpels, separate pipettes, and separate fresh reagents to avoid cross contamination. The LU_sediment samples (1 g each) and the membrane filters were subjected to DNA extraction by using the MoBio PowerSoil^®^ DNA Isolation Kit (MoBio Laboratories Inc., Carlsbad, CA, USA; cat # 12888–100). Briefly, each sample was transferred into separate 2 mL PowerBead tubes and then used for community DNA extraction. The purified DNA in triplicate was pooled into a single sample to obtain enough DNA that collectively represented the microbial community composition in the LU_water and LU_sediment samples [37,38]. The quality and concentration of the pooled DNA from each water and sediment sample was determined by using a Lambda II spectrophotometer (Perkin Elmer, Norwalk, Conn.) followed by agarose gel electrophoresis (1% wt/vol agarose in 1X Tris-Acetate-EDTA (TAE) buffer, pH 7.8) [39]. Then, the purified DNA samples were dried in a Savant Speedvac Evaporator SVC 100H and stored at 4 °C until further use for NGS. All prepared samples were subjected to shotgun metagenomics sequencing on the Illumina HiSeq platform (paired-end, 2 by 101 bp) at the UAB Heflin Center for Genomic Science (http://www.uab.edu/hcgs/).

### 2.3. Sequence Reads Processing Using Bioinformatics Tools

Raw sequence reads from the LU_water and LU_sediment samples were quality-checked and then filtered to remove sequence reads shorter than 50 bp and reads with an average quality score less than phred 20 using FASTX-Toolkit (http://hannonlab.cshl.edu/fastx_toolkit/), SICKLE [45], and FastQC [46]. The filtered sequences were then assembled into contigs using IDBA-UD [47] with default parameters followed by assembly quality checking using QUAST [48]. For protein annotation, the resulting sets of contigs were submitted to MG-RAST using their default quality control parameters and subjected to a similarity search using the SEED database [49] keeping 10^−5^ as the maximum E-value.

The other seven publicly available Antarctic metagenome datasets used in this study (Table 1) were previously sequenced, preprocessed, assembled, and uploaded into the MG-RAST server by other investigators (Table 1). The specific sequence analysis and/or processing information for these datasets was reported by other investigators (see references in Table 1).

### 2.4. Filtering Cold-Induced and Cold-Associated General Stress-Responsive Proteins Using R Code

All annotated metagenomics datasets, including LU_water and LU_sediment, were used to filter a total of 36 CIPs and CASPs (Table 2). To filter the aforementioned protein sequences from these metagenomics datasets, a user-defined R code [18] was used with specific parameters (>10 alignment lengths, >65% sequence identity to a subsystem, and E-value ≤ 10^−5^). After filtering, the total number of CIPs and CASPs was listed using Microsoft Excel software (Microsoft, Seattle, WA, USA) (Table 2).

### 2.5. Comparison of the Taxonomic Distribution and the Filtered Protein Sequences Using Bioinformatics Tools

Taxonomic profiles at the phylum level, including the domains Archaea, Bacteria, Eukaryota, and viruses across all samples, were assigned against the SEED database [49] using MG-RAST with specific parameters (>10 alignment lengths, >65% sequence identity to a subsystem, and E-value ≤ 10^−5^) and then downloaded for further analyses. The distribution and abundance of all taxonomic information was then visualized in a stacked column bar graph using Microsoft Excel software (Microsoft, Seattle, WA, USA.). In order to obtain certain taxa (at the genus level) contributing to statistical significance variation between the combined soil and lake sediment group (MS_soil, MDV_soil, LU_sediment) and the water group (LU_water, AL_water, and NB_water), we conducted a two-sided Welch’s *t*-test [50] with no correction and 95% confidence intervals with default parameters and then visualized the results in an extended error plot using STAMP analytical software [51].

Distribution and abundance of the filtered CIPs and CASPs across all samples were compared by constructing multidimensional-scaling (MDS) plots [52,53,54]. Subsequently, a complete linkage hierarchical clustering dendrogram [53,55,56] was constructed following the Bray–Curtis similarity values [57] using PRIMER-6 software (Primer-E Ltd., Ply-mouth Marine Laboratory, Plymouth UK, v6.1.2). Multiple group comparison of all filtered CIPs and CASPs along with their upper hierarchical SEED categories was carried out through the heatmap function implemented in STAMP [51] along with the average neighbor (UPGMA) method with default parameters.

## 3. Results

### 3.1. Total Sequence Reads

Shotgun metagenomics sequencing resulted in a total of 23,981,221 raw sequence reads from the LU_water metagenome and 14,895,305 from the LU_sediment metagenome. Quality assessment and trimming processes produced 23,245,003 sequences reads from LU_water and 14,399,372 from LU_sediment. Preprocessed sequence reads were assembled and annotated, which resulted in a total of 96,838 sequence reads from the LU_water samples and 119,907 from the LU_sediment samples (Table 1). For the publicly available metagenomes, the previously annotated sequence reads of 114,319 from AL_water, 80,924 from NB_water, 91,656 from MS_soil, and 95,228 from MDV_soil were used (Table 1).

### 3.2. Taxonomic Distribution and Abundance

At the domain level, gene sequences were mostly assigned to domain Bacteria (ranging from 93 to 98%) in all Antarctic metagenomes (data not shown). Less than 1% of the total gene sequences were assigned as Eukaryota and viruses in all Antarctic metagenomes used in this study, except for AL_water (1.9% for eukaryotes; 3.8% for viruses). Archaea accounted for ~2% in LU_water and NB_water; ~1% in MS_soil and MDV_soil; and less than 1% in LU_sediment and AL_water of the respective metagenome sequences. Additionally, 0.29% of the total sequence reads in the MS_soil sample did not match to any known taxa.

**Figure 2:**
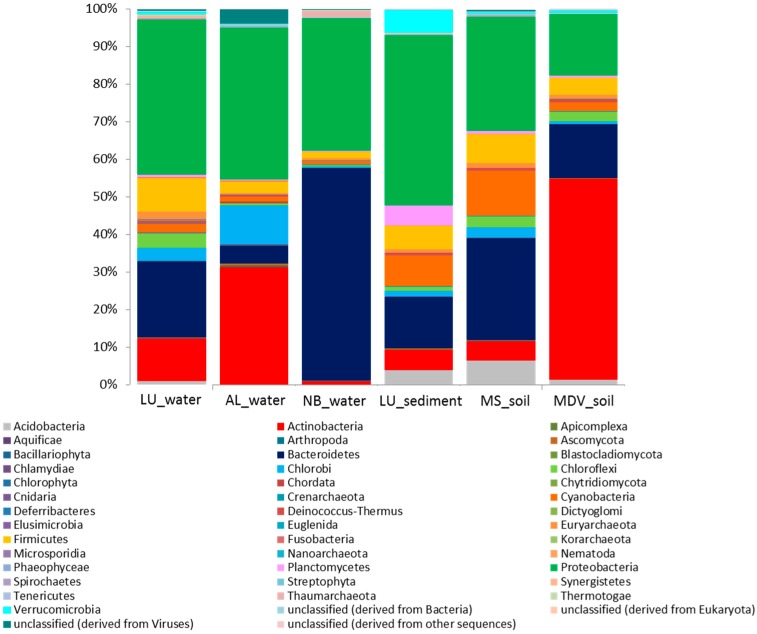
Stacked column bar graph representing the microbial community composition at the phylum level across all samples used in this study. Taxonomic identities that could not be shown to the respective level of resolution were considered as “unclassified” within their corresponding domain. Relative abundance data was analyzed by using MG-RAST against the SEED database and then visualized using Microsoft Excel Software (Microsoft, Seattle, WA, USA). Sample names are included in the plot (LU_water = Lake Untersee water; AL_water = Ace Lake water; NB_water = Newcomb Bay Lake water; LU_sediment = Lake Untersee sediment; MS_soil = Mount Seuss soil; MDV_soil = McMurdo Dry Valleys soil).

The relative abundances of microbial taxa at the phylum level showed that Proteobacteria was considerably abundant in all Antarctic metagenomes, ranging from 16.3 to 45.3% (Figure 2). In contrast, Bacteroidetes and Actinobacteria appeared to be the most abundant phylum in the NB_water (56.4%) and MDV_soil (53.3%) samples, respectively.

**Figure 3:**
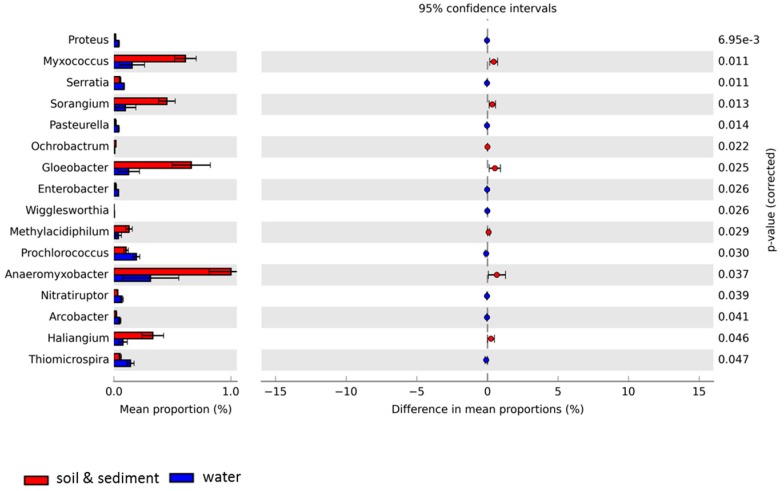
Extended error plot for taxonomic compositions at the genus level for Antarctic metagenomes used in this study was visualized through STAMP based on SEED subsystems. Total mean proportions in different categories are displayed in the left bar graph; the upper side (red) indicates the soil and lake sediment group (Lake Untersee sediment, Mount Seuss soil, and McMurdo Dry Valleys soil), and the lower side (blue) represents the water group (Lake Untersee water, Ace Lake water, Newcomb Bay Lake water). The colored circles (red and blue) show the 95% confidence intervals calculated using the Welch’s *t*-test [50] with no correction and default parameters.

The microbial profiles in all Antarctic samples at the genus level showed diverse microbial taxa, with similarities but also significant differences, particularly between the water group (LU_water, AL_water, and NB_water) and the combined soil (MS_soil and MDV_soil) and lake sediment (LU_sediment) group (Figure 3). Overall, *Myxococcus*, *Gloeobacter*, *Anaeromyxobacter*, and *Haliangium* made a significant contribution to the combined soil and lake sediment metagenomes as compared to the water metagenomes. Conversely, *Prochlorococcus* and *Thiomicrospira* made a more significant contribution to the taxonomic profiles of the water metagenomes than the combined soil and lake sediment metagenomes.

### 3.3. Comparative Analyses of Functional Profiles

**Figure 4:**
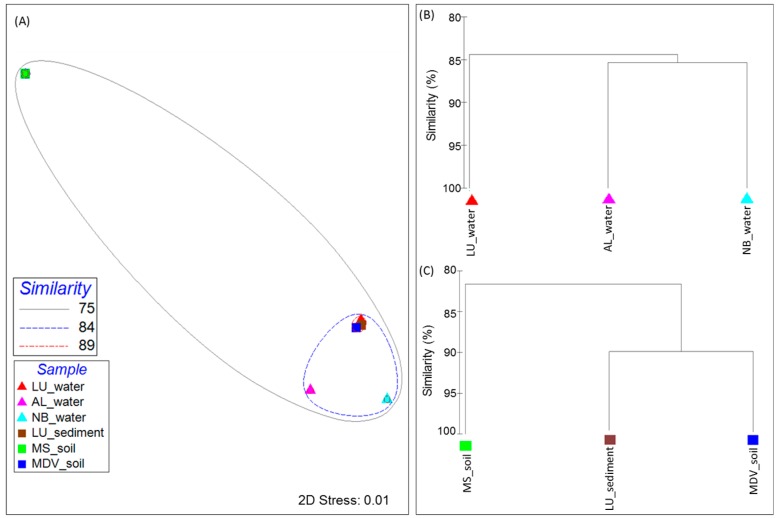
Beta diversity visualization of the filtered cold-induced and cold-associated general stress-responsive proteins across all samples used in this study. (**A**) Multidimensional-scaling (MDS) plot analysis representing the cluster pattern in a two-dimensional (2D) rendering with similarity overlays. Samples are denoted as triangles and rectangles for the sample group type (corresponding to the water group and the soil and lake sediment group, respectively) in the plot, and are colored by the sample. The dendrogram displays the cluster patterns of (**B**) the water and (**C**) soil and lake sediment group samples according to the Bray–Curtis value as a percentage (0 to 100). Both plots were generated through PRIMER-6 Ecological Software based on the Bray–Curtis similarity metric. Sample names are included in the plot (LU_water = Lake Untersee water; AL_water = Ace Lake water; NB_water = Newcomb Bay Lake water; LU_sediment = Lake Untersee sediment; MS_soil = Mount Seuss soil; MDV_soil = McMurdo Dry Valleys soil).

The distribution and relative abundance of genes involved in CIPs and CASPs found in all metagenomes used in this study showed distinct clustering patterns among the samples (Figure 4). The MDS plots of the LU_water, LU_sediment, and MDV_soil samples were clustered together at 89% Bray–Curtis similarity (Figure 4A). All samples, except MS_soil, grouped together at 84% Bray–Curtis similarity. The MS_soil sample showed relatively higher intra-sample variability, although it did cluster together with all other samples at 75% Bray–Curtis similarity. These clustering patterns were supported by the complete linkage hierarchical clustering dendrogram analysis (Figure 4B,C). Within the three water samples, LU_water revealed a slight intra-group variability as compared to the other two water samples (AL_water and NB_water) (Figure 4B). Among the soil and lake sediment group, LU_sediment showed a high similarity with MDV_soil; however, these two samples were observed to be separated from the MS_soil.

### 3.4. Cold-Induced Proteins in the Water Metagenomes

**Figure 5:**
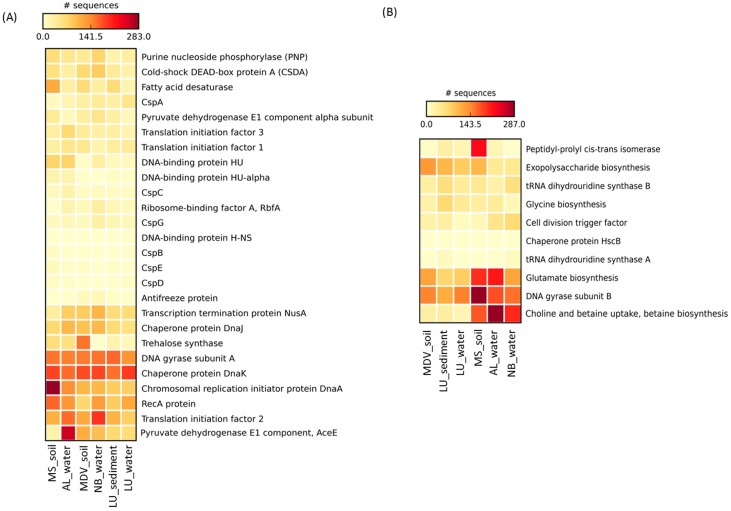
Heatmap representing the relative abundance of the (**A**) cold-induced proteins and (**B**) cold-associated general stress-responsive proteins found in this study against the SEED database. The heatmap was generated through STAMP [51] with the average neighbor (UPGMA) method using the default parameters. Larger values are represented in red and the smaller values in light yellow. Sample names are included in the plot (LU_water = Lake Untersee water; AL_water = Ace Lake water; NB_water = Newcomb Bay Lake water; LU_Sediment = Lake Untersee sediment; MS_soil = Mount Seuss soil; MDV_soil = McMurdo Dry Valleys soil).

In the LU_water metagenome, all 26 CIPs were detected, showing a high number of genes (>100 sequences) associated with chaperone protein DnaK, DNA gyrase subunit A (GyrA), and general recombination and DNA repair protein (RecA) (Figure 5A and Table 2). Interestingly, as compared to the AL_water and NB_water samples, the CspB and H-NS proteins were only found in LU_water samples.

In the AL_water metagenome, a total of 24 CIPs were found, showing a relatively high abundance of genes (>100 sequences) related to pyruvate dehydrogenase E1 component (AceE), chaperone proteins (DnaK and DnaJ), translation initiation factor 2 (IF2), GyrA, chromosomal replication initiator protein (DnaA), and RecA (Figure 5A and Table 2). As compared to LU_water and the NB_water, AL_water showed >2.5 times higher sequence reads for the pyruvate dehydrogenase E1 component protein (AceE).

In the NB_water metagenome, a total of 21 CIPs were found, showing a high number of genes (>100 sequences) related to IF2, DnaK, GyrA, RecA, transcription termination protein (NusA), DnaA, and DnaJ (Figure 5A and Table 2). Interestingly, purine nucleoside phosphorylase (PNP) and cold-shock DEAD-box protein A (CSDA) were found to be >1.5 times higher in the NB_water as compared to the AL_water and LU_water metagenomes.

Overall, gene sequences associated with GyrA, RecA, DnaA, DnaJ, DnaK, IF2, and NusA were found to be predominant in all three water metagenomes.

### 3.5. Cold-Induced Proteins in the Combined Soil and Lake Sediment Metagenomes

All 26 CIPs were found in the LU_sediment metagenome, revealing a high number of genes (>100 sequences) associated with IF2, GyrA, and DnaK (Figure 5A and Table 2). Interestingly, H-NS was only found in LU_sediment as compared to the MS_soil and MDV_soil metagenomes.

A total of 25 CIPs were detected in the MS_soil metagenome, revealing a high number of genes (>100 sequences) associated with DnaA, DnaK, RecA, GyrA, fatty acid desaturase (FAD), and IF2 (Figure 5A and Table 2). Particularly, RecA, DnaA, PNP, FAD, and a DNA-binding protein (HU) were considerably higher in the MS_soil as compared to the LU_sediment and the MDV_soil metagenomes.

In the MDV_soil metagenomes, a total 24 CIPs were found, among which DnaK, trehalose synthase (TS), GyrA, IF2, AceE, and DnaA were highly abundant (>100 sequences) (Figure 5A and Table 2). As compared to the LU_sediment and MS_soil metagenomes, TS and AceE were substantially abundant in MDV_soil.

In general, gene sequences for the DnaK, GyrA, IF2, RecA, DnaA, and FAD proteins were highly abundant in the combined soil and lake sediment metagenomes.

### 3.6. Cold-Associated General Stress-Responsive Proteins in the Water Metagenomes

All 10 CASPs were found in the LU-water metagenome, showing DNA gyrase subunit B (GyrB) as the most abundant (>100 sequences), followed by exopolysaccharide (EPS) biosynthesis (85 sequences) and glutamate biosynthesis (83 reads) (Figure 5B and Table 2). Especially, EPS biosynthesis was more abundant (~2 times more) in the LU_water than the AL_water and the NB_water metagenomes.

A total of 9 CASPs were found in the AL_water metagenome, showing choline and betaine uptake and biosynthesis and glutamate biosynthesis as the most highly abundant CASPs (>200 sequences) followed by GyrB (178 sequences) (Figure 5B and Table 2). Additionally, choline and betaine uptake and biosynthesis and glutamate biosynthesis sequences were found to be the most abundant in the AL_water metagenome among all water metagenomes used in this study.

A total of 10 CASPs were detected in the NB_water metagenome. Choline and betaine uptake and biosynthesis were the most abundant CASPs in the NB_water metagenome followed by GyrB (160 reads) and glutamate biosynthesis (119 reads) (Figure 5B and Table 2). A relatively higher (>1.5 times more) abundance of both tRNA dihydrouridine synthase A and B was found in the NB_water metagenome compared with the AL_water and LU_water metagenomes.

In general, as compared to the LU_water metagenome, the distribution of CASPs, particularly the gene sequences for glutamate biosynthesis (214 and 119 reads, respectively) and choline and betaine uptake and biosynthesis (287 and 204 reads, respectively), was highly abundant in the AL_water and NB_water metagenomes. GyrB was highly abundant across all water metagenomes.

### 3.7. Cold-Associated General Stress-Responsive Proteins in the Combined Soil and Lake Sediment Metagenomes

In the LU_sediment metagenome, a total of 10 CASPs were found, revealing GyrB and EPS biosynthesis as the most abundant CASPs (>100 sequences) (Figure 5B and Table 2). Genes related to both tRNA dihydrouridine synthase A and B were relatively higher (75 total reads) in the LU_sediment metagenome than in the MS_soil (43 reads) and MDV_soil (32 reads) soil metagenomes.

A total of 9 CASPs were found in the MS_soil metagenome, in which GyrB sequences were found to be the most abundant (>200 sequences) followed by peptidyl-prolyl cis-trans isomerase, glutamate biosynthesis, choline and betaine uptake and biosynthesis, and EPS biosynthesis (Figure 5B and Table 2). All of these sequences were also substantially abundant in the LU_sediment and MDV_soil metagenomes.

In the MDV_soil metagenome, a total of 8 CASPs were identified, in which GyrB was the most abundant (>200 sequences) followed by EPS biosynthesis (131 reads) and glutamate biosynthesis (123 reads) (Figure 5B and Table 2). Interestingly, chaperone protein HscB and tRNA dihydrouridine synthase A were absent in the MDV_soil metagenome when compared to the LU_sediment metagenome.

Overall, glutamate biosynthesis, GyrB, and EPS biosynthesis were the highly abundant CASPs across all combined soil and lake sediment metagenomes.

## 4. Discussion

The rapid advancements of culture-independent NGS have revolutionized our understanding of the microbial communities and their functional genes in a wide range of ecosystems, including the polar environments [58,59,60]. By using this approach, the bacterial metabolic genes for adaptation to cold temperature environments have been studied in cyanobacterial mats in Arctic and Antarctic ice shelves [21], microbial mats from Antarctic Lake Joyce [18], and permafrost samples from Alaska [61]. In order to obtain collective insights into the microbial distributions and abundances of genes associated with CIPs and CASPs, we have analyzed metagenomes from Lake Untersee water and sediment samples for comparison with selected publicly available soils and water metagenomes from diverse ecosystems in the Antarctic continent.

In general, the microbiota of all metagenomes used in this study showed mostly comparable taxonomic compositions. For example, Proteobacteria and Bacteroidetes were the abundant phyla, whereas phylum Actinobacteria, although varied in their abundances, were found in all metagenomes. The microbial taxa and cold-adaptive traits found in our samples have also been reported previously in diverse Antarctic soil, sediment, and aquatic ecosystems [21,62,63,64,65]. Despite the similarities in microbial composition across all metagenomes, noticeable differences at the genus level were found when compared between the water and the combined soil and lake sediment metagenomes. The water metagenomes had relatively higher abundances of *Prochlorococcus* and *Thiomicrospira* than the soil and lake sediment metagenomes, which was also reported in several sub-zero Antarctic lakes [15]. *Prochlorococcus* and *Thiomicrospira* are known to be one of the key contributors to Antarctic aquatic ecosystems as they are characterized as photosynthetic organisms and autotrophic sulfur-oxidizing gammaproteobacterium, respectively [15]. In contrast, the soil and lake sediment metagenomes showed relatively higher abundances of *Myxococcus*, *Anaeromyxobacter,* and *Haliangium* than the water metagenomes. *Myxococcus*, *Anaeromyxobacter,* and *Haliangium* have been previously found in other Antarctic soil samples [66]; and *Gloeobacter* has been reported in Antarctic sediment samples [67]. Interestingly, myxobacteria (such as *Myxococcus*) have been generally considered to be mesophilic soil microbes [68]; however, the first psychrophilic myxobacteria were identified in soil samples in Antarctic McMurdo Dry Valleys and South Victoria Land [68]. A few other studies have also reported *Anaeromyxobacter, Haliangium,* and *Gloeobacter* in Antarctic soil and sediment ecosystems.

The cold-adaptive traits in water metagenomes revealed a generally similar distribution of CIPs, including a high number of genes associated with DNA replication (GyrA, RecA, and DnaA), protein folding (chaperone proteins DnaJ and DnaK), protein biosynthesis (translation initiation factor), and the transcription termination protein NusA. Like the water metagenomes, the soil and lake sediment metagenomes also showed similar CIP distributions in all samples. Within the cold stress proteins (cold shock family of proteins), CspA was found to be highly abundant in each water and combined soil and lake sediment metagenome. At low temperatures, cold stress proteins are expressed quickly and remain active to stabilize the mRNA, thus helping proper protein folding and allowing bacteria to adapt their physiology to the cold temperature environments [17,18,21,69,70]. Particularly, CspA is known to function as an RNA chaperone, destabilizing the secondary structures of mRNA necessary for the expression of the cold-inducible proteins and enhancing the expression of GyrA [18,71,72]. Moreover, CspA, CspC, and CspE act as transcriptional anti-terminators, allowing alternative mechanisms for the regulation of other CIPs, such as NusA, IF2, RbfA, and PNPase [73]. Although each water and combined soil and lake sediment metagenome showed a lower abundance of cold-responsive stress proteins as compared to DnaA, DnaK, DnaJ, DNA topoisomerases, and recombination factors, these proteins have been observed to be highly abundant, particularly in Antarctic and Arctic ecosystems. This is due to their role in helping bacteria maintain steady-state cellular metabolism, growth, and division in order to cope with the consistent cold environments [18,21].

In the presence of cold stress, bacterial cell membranes undergo decreased membrane fluidity but an increase in permeability. It has been reported that FAD offsets membrane stiffness by modifying the existing fatty acid chain structures of the cell membrane [74,75,76,77]. Moreover, TS has been characterized to be involved in numerous stress-related processes and predicted to function in the restriction of oxidative damage, cryopreservation, and cell membrane protection [78,79,80,81]. The combined soil and lake sediment metagenomes showed more genes related to FAD and TS than the water metagenomes. Especially, FAD and TS were more abundant in the MS_soil and the MDV_soil metagenomes, implying that protective responses are needed for bacteria surviving in the open soil ecosystems due to the fluctuations in temperatures, desiccation, and poor nutrient availability. H-NS is known as a nucleoid-associated DNA binding protein [82] and a regulator of the expression of various cold shock genes [83,84,85,86]. In our study, H-NS was only found in the LU_water and the LU_sediment metagenomes. This may support the presence of almost the entire cold-shock family of proteins (CspA, CspB, CspC, CspD, CspE, CspG, and antifreeze proteins) in the metagenomes of Lake Untersee.

The distribution of CASPs in each water and combined soil and lake sediment metagenome showed genes associated with the regulation of GyrB (DNA gyrase) and glutamate biosynthesis. DNA gyrase is known to play an important role in regulating DNA topology during transcription and manifests higher activity at cold temperatures [73,77,87]. Glutamate, glycine, choline, and betaine are known cryo- and osmoprotectants [21,88], thus supporting our results of heightened glutamate synthesis genes observed in all metagenomes. Furthermore, choline and betaine biosynthesis allow bacteria to increase osmolality, thus helping to protect against cold-related damage to the cell structure and function [89]. A high representation of glutamate biosynthesis in all metagenomes used in this study might reflect the high osmotic stress present across the Antarctic continent. Additionally, the high number of genes associated with choline and betaine biosynthesis found in the AL_water and NB_water metagenomes as opposed to the LU_water metagenome may be due to the relatively higher salt concentrations in the Ace and Newcomb Bay lakes as compared to the freshwater of Lake Untersee. EPS also plays an important role in cryoprotection against ice crystal damage and high salinity [19,21]. A relatively higher abundance of genes associated with EPS biosynthesis was found in Lake Untersee and the combined soil and lake sediment metagenomes than in Ace Lake and Newcomb Bay Lake metagenomes. This may be due to a relatively higher abundance of Cyanobacteria, which are known to produce a copious amount of EPS [21]. All water and combined soil and lake sediment metagenomes had a noteworthy distribution of tRNA dihydrouridine synthase, which is known to help maintain conformational flexibility and dynamic motion of tRNA at cold temperatures [90]. Thus, an abundance of sequences for tRNA dihydrouridine synthase in our metagenomes indicates an adaptive advantage in microorganisms inhabiting the Antarctic environment.

In a previous study, the shotgun metagenomics approach was applied to the microbial communities of a laboratory culture of *Euplotes focardii*, a psychrophilic marine ciliate collected from sediments in Terra Nova Bay, Antarctica [91]. These microbial communities were considered to be representative of the Antarctic sample upon collection, and, similar to this study, showed a heightened distribution of the phyla Proteobacteria followed by Bacteroidetes. Functional analysis demonstrated ice binding and antifreeze proteins and proteins involved in the oxidative stress response, which supported the postulated underlying genetic capacity for adaptation to their consistently cold and oxygen-rich environment. Interestingly, antibiotic treatment of the ciliate cultures showed a reduction in the proliferation of *E. focardii*, which was attributed to the loss of key biogeochemical (carbon and nitrogen) and nutrient cycling performed by the associated microbiota. As such, the various cold-responsive stress genes (CIPs and CASPs) observed in the extreme Antarctic ecosystems of this study demonstrate crucial microbial adaptations to cold stress, allowing for both their persistence and possible sustenance of other inhabiting organisms that are metabolically restricted by the cold stress.

The mechanisms of bacterial genetic adaptation in low- and subzero-temperature environments have been well-reported [63,69]. Our analyses included the updated list of CIPs and CASPs found in microbial metagenomes. These proteins have been filtered from the metagenomics datasets by using bioinformatics tools to achieve a comprehensive outlook of microbial community composition and mechanisms to cope with cold and other stresses present in Antarctica. Overall, noticeable differences were found in the microbial taxa distribution and various cold- and stress-related functions among all Antarctic metagenomes. However, the key genes necessary for adaptation in the continuous low- and subzero-temperature environment were well-represented across all Antarctic metagenomes used in this study. Moreover, the permanently ice-covered Lake Untersee metagenomes had high abundances of sequences for cold-responsive stress proteins and H-NS, indicating that this lake environment poses comparatively greater survival challenges to the inhabiting microbial communities.

## Figures and Tables

**Table 1:** Antarctic metagenomics sample information used in this study. Included in the table is the location of sampling, sample type reflecting the environment of the collection, the total annotated sequence reads in each sample found in the Metagenomics Analysis Server (MG-RAST), and the MG-RAST ID number.

Samples	Location	Sample Type	Total Annotated Sequence Reads	MG-RAST ID	Reference
Lake Untersee	71.35609° S 13.4268° E	Freshwater	96,838	This study	This study
Ace Lake	68.47° S 78.18° E	Saline water (originally freshwater)	114,319	mgm4443684.3	[40,41]
Newcomb Bay Lake	66.27° S 110.53° E	Marine habitat water	80,924	mgm4443686.3	[42]
Lake Untersee	71.34197° S 13.45458° E	Sediment	119,907	This study	This study
Mount Seuss	77.02° S 161.85° E	Soil	91,656	mgm4667023.3	[43]
McMurdo Dry Valleys	77.63° S 162.88° E ^1^	Soil	27,582	mgm4575389.3	[44]
	77.73° S 162.31° E ^2^	Soil	25,064	mgm4575387.3	
	77.60° S 163.25° E ^3^	Soil	18,398	mgm4575388.3	
	77.53° S 161.70° E ^4^	Soil	24,184	mgm4575390.3	

^1^ Taylor Valley floor adjacent to Lake Hoare; ^2^ Taylor Valley floor adjacent to Lake Bonney; ^3^ Taylor Valley floor adjacent to Lake Fryxell; and ^4^ Wright Valley.

**Table 2:** Number of genes related to the cold-induced proteins and cold-associated general stress proteins found across all Antarctic metagenomes used in this study.

	Number of Genes Found in Each Antarctic Metagenome					
**Protein or SEED subsystem name**	**Lake Untersee water**	**Ace Lake water**	**Newcomb Bay water**	**Lake Untersee sediment**	**Mount Seuss soil**	**McMurdo Dry Valleys soil**
**Cold-Induced Proteins**						
Pyruvate metabolism						
Pyruvate dehydrogenase E1 component, AceE	65	255	96	70	26	111
Pyruvate dehydrogenase E1 component alpha subunit	13	12	50	33	39	35
Cold stress proteins						
CspA	49	21	40	31	11	33
CspB	2	0	0	1	8	0
CspC	8	26	9	8	11	8
CspD	7	4	0	3	8	1
CspE	2	4	1	4	12	5
CspG	15	15	29	18	11	12
Antifreeze protein	3	3	15	6	1	10
Di- and oligosaccharides						
Trehalose synthase	14	58	0	24	62	156
Protein biosynthesis						
Translation initiation factor 1	38	49	23	36	31	46
Translation initiation factor 2	80	160	193	106	108	114
Translation initiation factor 3	23	68	29	33	33	39
Ribosome-binding factor A, RbfA	11	26	35	17	3	14
Clustering-based subsystems						
Transcription termination protein NusA	65	81	103	65	36	84
DNA metabolism						
DNA-binding protein H-NS	3	0	0	2	0	0
DNA-binding protein HU	12	76	32	12	76	6
DNA-binding protein HU-alpha	4	23	1	7	23	3
DNA replication						
DNA gyrase subunit A	133	149	158	163	155	152
RecA protein	117	132	125	83	164	73
Chromosomal replication initiator protein DnaA	80	139	102	84	283	102
Nucleosides and nucleotides						
Purine nucleoside phosphorylase (PNP)	33	47	75	24	63	42
Protein folding						
Chaperone protein DnaJ	58	100	100	69	70	91
Chaperone protein DnaK	189	160	182	158	183	179
RNA metabolism						
Cold-shock DEAD-box protein A (CSDA)	27	31	84	40	58	70
Unsaturated fatty acids						
Fatty acid desaturase	22	33	34	66	114	65
**Cold-Associated General Stress-Responsive Proteins**						
Amino acids and derivatives						
Glycine biosynthesis	42	40	16	69	34	28
Glutamate biosynthesis	83	214	119	75	199	123
Bacterial cell division						
Cell division trigger factor	9	52	68	31	15	27
Osmotic stress						
Choline and betaine uptake, betaine biosynthesis	21	287	204	35	176	31
Cell wall and capsule (Capsular and extracellular polysaccharides)						
Exopolysaccharide biosynthesis	85	42	44	104	102	131
DNA replication						
DNA gyrase subunit B	150	178	160	115	286	149
Protein folding						
Peptidyl-prolyl cis-trans isomerase	18	18	3	31	224	5
RNA metabolism						
Chaperone protein HscB	1	0	1	3	2	0
tRNA dihydrouridine synthase A	1	3	9	12	0	0
tRNA dihydrouridine synthase B	40	28	60	63	43	32
